# Implantable Cardioverter Defibrillator mHealth App for Physician Referrals and eHealth Education: ICD-TEACH Pilot Study

**DOI:** 10.2196/10499

**Published:** 2018-11-05

**Authors:** Sumeet Gandhi, Carlos A Morillo, Jon-David Schwalm

**Affiliations:** 1 Population Health Research Institute Hamilton, ON Canada; 2 Hamilton Health Sciences Hamilton, ON Canada; 3 McMaster University Hamilton, ON Canada; 4 Libin Cardiovascular Institute of Alberta University of Calgary Calgary, ON Canada

**Keywords:** mHealth, smartphone app, implantable defibrillator cardioverter, ICD, physician decision, eHealth, mobile phone

## Abstract

**Background:**

Mobile health (mHealth) decision tools for implantable cardioverter defibrillator may increase physician knowledge and overall patient care.

**Objective:**

The goals of the ICD-TEACH pilot study were to design a smartphone app or mHealth technology with a novel physician decision support algorithm, implement a direct referral mechanism for device implantation from the app, and assess its overall usability and feasibility with physicians involved in the care of patients with an implantable cardioverter defibrillator.

**Methods:**

The initial design and development of the mHealth or smartphone app included strategic collaboration from an information technology company and key stakeholders including arrhythmia specialists (electrophysiologists), general cardiologists, and key members of the hospital administrative team. A convenience sampling method was used to recruit general internists or cardiologists that refer to our local tertiary care center. Physicians were asked to incorporate the mHealth app in daily clinical practice and avail the decision support algorithm and direct referral feature to the arrhythmia clinic. Feasibility assessment, in the form of a physician survey, was conducted after initial mHealth app use (within 3 months) addressing the physicians’ overall satisfaction with the app, compliance, and reason for noncompliance; usability assessment of the mHealth app was addressed in the physician survey for technical or hardware problems encountered while using the app and suggestions on improvement.

**Results:**

A total of 17 physicians agreed to participate in the pilot study with 100% poststudy survey response rate. Physicians worked in an academic practice, which included both inpatient and ambulatory care. System Usability Scale was applied with an average score of 77 including the 17 participants (>68 points is above average). Regarding the novel physician decision support algorithm for implantable cardioverter defibrillator referral, 11% (1/9) strongly agreed and 78% (7/9) agreed that the algorithm for device eligibility was easy to use. Only 1 patient was referred through the direct referral system via the mHealth app during the pilot study of 3 months. Feasibility assessment showed that 46% (5/11) strongly agreed and 55% (6/11) agreed that the mHealth app would be utilized if integrated into an electronic medical record (EMR) where data are automatically sent to the referring arrhythmia clinic.

**Conclusions:**

The ICD-TEACH pilot study revealed high usability features of a physician decision support algorithm; however, we received only 1 direct referral through our app despite supportive feedback. A specific reason from our physician survey included the lack of integration into an EMR. Future studies should continue to systematically evaluate smartphone apps in cardiology to assess usability, feasibility, and strategies to integrate into daily workflow.

## Introduction

**Figure 1:**
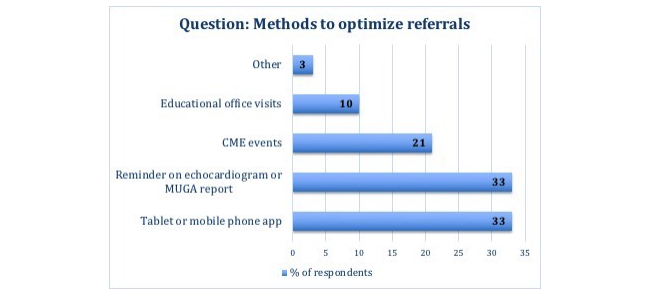
Questionnaire results. CME: continuing medical education; MUGA: multigated acquisition.

Guideline-recommended primary and secondary prevention of sudden cardiac death in high-risk patients includes placing an implantable cardioverter defibrillator in these patients [1,2]. Despite continuing medical education and physician-based interventions, there still remains a large population of eligible patients who may not be receiving such therapy [3]. Identifiable reasons for the lower than expected physician implantable cardioverter defibrillator referral rate have been attributed to misperception about the benefit of implantable cardioverter defibrillator therapy and patient eligibility, as well as the lack of awareness about the device implantation process. To understand the barriers of knowledge and potentially minimize care gaps that exist between evidence-based recommendations and current practice for implantable cardioverter defibrillator referral, a Web-based questionnaire was conducted predominantly including community-based family physicians and general internists. In this small sample of 24 physicians, 42% (10/24) of the participants were not familiar with current implantable cardioverter defibrillator guidelines, while a small number also believed implantable cardioverter defibrillator therapy did not improve quality of life. When asked about different methods to optimize referrals, a tablet or mobile phone app to help identify potential patients as well as reminders on echocardiograms or multigated acquisition report were highly selected (Figure 1).

Smartphone apps or mobile health (mHealth) technology are part of daily life, with continued growth gaining popularity among health care providers. Incorporated into the daily lives of both physicians and patients, mHealth has the ability to provide evidence-based guidance in a Web-based, engaging, and user-friendly format with instant knowledge acquisition [4]. As an adjunct to behavior modeling, the intervention of mHealth has demonstrated early success in improving patient and physician outcomes [5]. The purpose of the ICD-TEACH study was to design a smartphone app or mHealth technology with a novel physician decision support algorithm, implement a direct referral mechanism for implantable cardioverter defibrillator implantation from the app, and assess its overall usability and feasibility with physicians involved in the care of these patients.

## Methods

**Figure 2:**
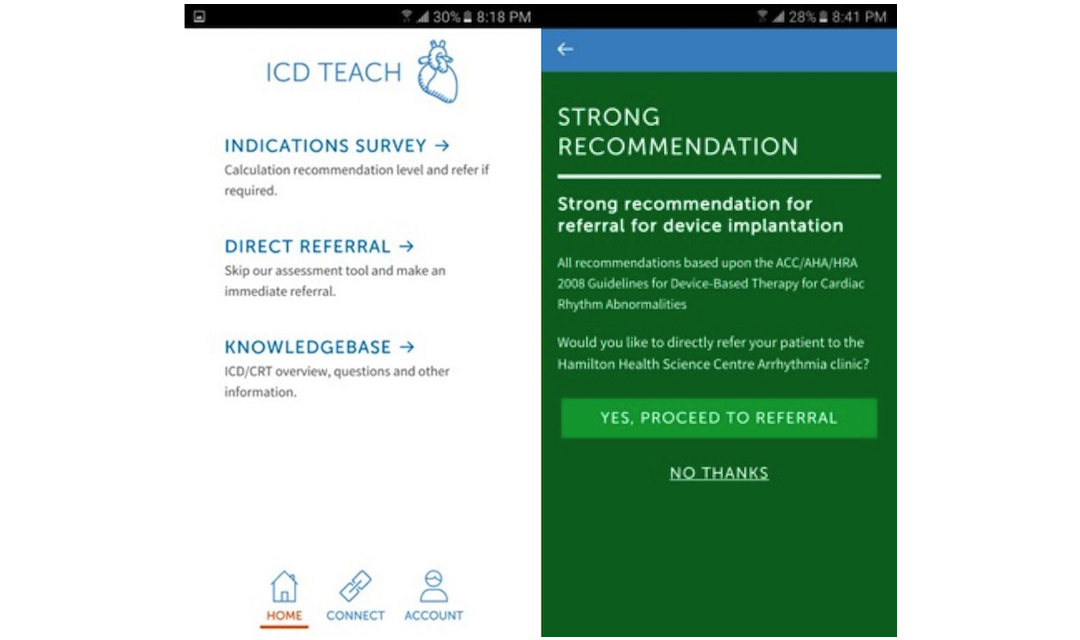
ICD-TEACH initial dashboard at log-in (left) and indication survey (right). Upon completing the indication survey, the algorithm provides a recommendation for implantable cardioverter defibrillator (based upon the Canadian Cardiovascular Society/Canadian Heart Rhythm Society 2016 Implantable Cardioverter-Defibrillator Guidelines). Users were also given the option to directly refer to the arrhythmia clinic.

ICD-TEACH was a single-center pilot study designed to assess the usability and feasibility of mHealth for implantable cardioverter defibrillator physician decision support and direct referral to a regional arrhythmia center. The initial design and development of the mHealth or smartphone app included strategic collaboration from an information technology company and key stakeholders including arrhythmia specialists (electrophysiologists), general cardiologists, and key members of the hospital administrative team. The mHealth app included an interactive, user-friendly algorithm to determine patients eligible for implantable cardioverter defibrillator implantation with instant feedback and the option for direct referral to our regional arrhythmia referral center in Ontario, Canada (Table 1). The mHealth app also provided education to physicians about ventricular arrhythmias, congestive heart failure, sudden cardiac death, procedure information, current guideline recommendations for device therapy, quality of life, day-to-day or frequently asked questions, and the ability to refer patients to a regional arrhythmia clinic, embedded within the app (Figure 2).

**Table 1:** Rule-based algorithm answered by the user to determine implantable cardioverter-defibrillator (ICD) indication. All recommendations based upon the Canadian Cardiovascular Society/Canadian Heart Rhythm Society 2016 Implantable Cardioverter-Defibrillator Guidelines.

Question		Strong recommendation		Weak recommendation	
		Rule 1	Rule 2	Rule 1	Rule 2
**Is the patient's left ventricular ejection fraction <55%?**					
	Yes	✓	✓	✓	✓
	No				
**The patient's ejection fraction is:**					
	>54%				
	36%-54%				
	31%-35%			✓	✓
	≤30%	✓	✓		
**Does the patient exhibit indications of:**					
	Ischemic heart disease or prior myocardial infarction	✓		✓	✓
	Nonischemic cardiomyopathy		✓		
	None of the above apply				
**Ischemic cardiomyopathy: Has at least 40 days passed since the most recent myocardial infarction or 3 months postrevascularization? Nonischemic cardiomyopathy: Has at least 3 months passed with the patient on optimal medical therapy?**					
	Yes	✓	✓	✓	✓
	No				
**Is the patient's expected survival with a good functional status ≥1 year?**					
	Yes	✓	✓	✓	✓
	No				
**The patient has familial or personal history of arrhythmogenic right ventricular dysplasia, Brugada syndrome, catecholaminergic polymorphic ventricular tachycardia, long QT syndrome, short QT syndrome, or hypertrophic cardiomyopathy?^a^**					
	Yes				
	No				

^a^Answer does not affect algorithm.

The next phase of this pilot study included rollout of the mHealth app to cardiology and internal medicine physicians to assess its usability and feasibility. A convenience sampling method was used to recruit general internists or cardiologists who refer patients to our local tertiary care center. Physicians were eligible to participate if their current practice pattern included patients with congestive heart failure and if they were current smartphone users (Apple-, Android-, or Blackberry- based platforms with access to mobile data).

**Figure 3:**
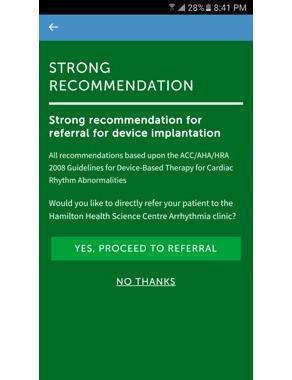
Direct referral: option to directly refer to the arrhythmia clinic.

Participating physicians were asked to independently review a document about the mHealth app and project goals. Instructions were provided to review the app content and assess patient eligibility for implantable cardioverter defibrillator therapy using the algorithm. Physicians were asked to incorporate the mHealth app in daily clinical practice and avail the decision support algorithm and direct referral feature to the arrhythmia clinic (Figure 3). A physician survey was conducted after initial mHealth app use (within 3 months) to assess physicians’ overall satisfaction with the app, compliance, reason for noncompliance, technical or hardware problems encountered while using the app, and suggestions on improvement. Reminders were provided via email at 1 week, 4 weeks, and 3 months to reiterate the benefit and promote the mHealth app.

The primary outcome of this study was to assess the feasibility of incorporating an mHealth app into daily clinical practice. A descriptive analysis was performed based on the structured questionnaire regarding satisfaction of completing the task, overall compliance, the reason for noncompliance, technical or hardware problems encountered while using the app, and suggestions on improvement. We also tracked the number of referrals to the regional arrhythmia service clinic through the app. Our usability assessment included the System Usability Scale incorporated into the survey, which has been validated for health care-related smartphone apps; this scale consists of specific questions evaluating mHealth technology with a 5-point Likert scale [6]. A score >68 points is above average and indicates adequate usability. The authors had full access to the data and take full responsibility for its integrity. The study was approved by the Hamilton Integrated Research Ethics Board (HIREB Project #15-208).

## Results

### Survey Results

Physician recruitment was performed from January 1 to 30, 2017. Participating physicians were able to use the app for a total of 3 months, and final survey results and the pilot study were completed by May 1, 2017. A total of 17 physicians agreed to participate in the study with a 100% survey response rate. Physicians worked in an academic practice, which included both inpatient and ambulatory care; 76% (13/17) participants were general cardiologists or residents and 26% (4/17) were general internal medicine specialists. Among the respondents, 14% (2/14) agreed that the current paper- or fax-based system for device referral was difficult, 29% (4/14) disagreed, and 57% (8/14) were neutral. Furthermore, 21% (3/14) agreed that they enjoyed using the current system for implantable cardioverter defibrillator referral, while 21% (3/14) disagreed. Participating physicians thought the app was not difficult to download or install on the smartphone device and did not take too long to download.

**Figure 4:**
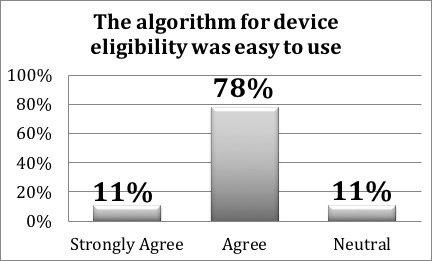
Poststudy survey results.

Regarding usability, the System Usability Scale was applied with an average score of 77 including the 17 participants (>68 points is above average). Furthermore, regarding the novel physician decision support algorithm for implantable cardioverter defibrillator referral, 11% (1/9) strongly agreed and 78% (7/9) agreed that the algorithm for device eligibility was easy to use (Figure 4). For the direct referral option to our regional arrhythmia center, 88% (7/8) agreed and 12% (1/8) strongly agreed that the direct referral process was also easy to use. When asked about whether they would use this mHealth app for direct referral, 25% (2/8) strongly agreed, 50% (4/8) agreed, 13% (1/8) disagreed, and 13% (1/8) were neutral. Respondents felt that the education material was beneficial such as procedure information (6/8, 75%, agreed and 2/8, 25%, strongly agreed), device therapy information (2/7, 29%, strongly agreed and 4/7, 57%, agreed), and common frequently asked questions (1/7, 14%, strongly agreed and 5/7, 71%, agreed). Meanwhile, 67% (8/12) disagreed that the current traditional paper- or fax-based system was more efficient, while 17% (2/12) agreed.

The majority of physicians felt that this mHealth app should be available to all physicians in the province of Ontario (3/7, 43%, strongly agreed and 3/7, 43%, agreed). Moreover, when asked whether entering patient information into the app was difficult, 9% (1/11) strongly disagreed and 46% (5/11) disagreed. When asked whether they did not trust or rely on the app to submit private information, 27% (3/11) disagreed and 9% (1/11) strongly disagreed, while 46% (5/11) were neutral, 9% (1/11) agreed and 9% (1/11) strongly agreed. Among the respondents, 18% (2/11) disagreed and 9% (1/11) strongly disagreed with the comment that they did not need an algorithm for the device when a patient needed an implantable cardioverter defibrillator, while 55% (6/11) were neutral and 18% (2/11) agreed or strongly agreed.

### Feasibility or Uptake of the mHealth App

Physician referrals using the mHealth app were tracked during the study period. Only one patient was referred through the direct referral system via the mHealth app during the pilot study of 3 months. Feasibility assessment showed that 46% (5/11) strongly agreed and 55% (6/11) agreed that mHealth app would be utilized if integrated into an electronic medical record (EMR) where data are automatically sent to the referring arrhythmia clinic. This was also reflected in the free-text comments provided by physicians at the end of the survey. Moreover, 91% (10/11) agreed and 9% (1/11) strongly agreed that the mHealth app should be available in a Web or browser format.

Further feedback included that during the pilot study, overall patient encounters (case numbers to use the mHealth tool) where patients needed to be referred for device implantation was low. Physicians also mentioned that in their current practice, it would be easier to fill out a referral form than submit through the app and that most cardiologists did not frequently need a decision support algorithm for device referral.

## Discussion

### Principal Findings

The results of the ICD-TEACH pilot study revealed that our novel implantable cardioverter defibrillator decision support algorithm and direct referral mechanism was easy to use with adequate usability. Physicians did not find entering patient information cumbersome, felt comfortable submitting patient information through the mHealth app, and believed that the algorithm tool should be disseminated widely. The education materials regarding procedural information, device therapy, guideline summaries, and frequently asked questions were useful and informative. Despite majority of the physicians stating that they would use the direct referral mechanism, we received only one direct referral through the mHealth app during the study period of 3 months.

Several challenges were faced in integrating this mHealth technology into the routine practice of physicians. We initially anticipated 40-50 physicians for enrollment into the pilot study; however, after recurrent contact through email, the response to the recruitment email (accept or not accept) was low. Although speculative, this may be due to the fact that physicians already have a current system that is efficient and did not want to spend additional time learning a new system if significant efficiency was not to be gained. This may be reflected in that we only received one direct referral through the mHealth app. Our survey results suggested that cardiologists did not need an algorithm for device implantation and that the current paper- or fax-based system was efficient enough for daily use. Our population of cardiologists and general internists who participated in this pilot study worked in a tertiary care or urban center; they may have different perceptions than physicians in rural settings, who may not have timely access to subspecialty referral. The ICD-TEACH app may offer more benefits to physicians practicing in rural areas, medical students or resident physicians, and primary care physicians looking for further education about implantable cardioverter defibrillator referral as well learning guideline-based indications for device referral through the algorithm.

One important insight gained from our pilot study through the survey and free-text comments is that the optimal utilization of an mHealth app with a decision support algorithm and direct referral mechanism should be linked directly to an EMR system. In this manner, once the decision to refer a patient for implantable cardioverter defibrillator is made, the EMR system would autopopulate the patient information fields and also send the appropriate information to the arrhythmia clinic (such as patient history, medications, blood work, and key investigations). A limitation of our pilot study is that our current health care network does not have an EMR or an electronic referral mechanism; we could have seen more direct referrals if the ICD-TEACH app was integrated into an EMR software.

With the rise of health care-related mHealth technology, it is important that the medical community evaluates such tools in a systematic manner. Cardiovascular societies and health care organizations should look to formally test health care mHealth apps for usability and feasibility to gain further insight and feedback, in the form of pilot studies or focus group testing. A goal of our pilot study was to assess the feasibility of incorporating a physician decision support tool for implantable cardioverter defibrillator through mHealth technology. Several pitfalls were highlighted, which clearly demonstrate that before mHealth technology integration, there has to be an incentive to increase efficiency as well as a platform for ease of access (such as EMRs) in order for a direct referral process to be successful. To the best of our knowledge, our objective usability assessment is one of the first to be tested on physician decision tools through mHealth. These findings will further allow us to make changes to the mHealth tool to optimize use in the future.

### Conclusion

The ICD-TEACH pilot study revealed high usability features of a physician decision support algorithm and direct referral mechanism for implantable cardioverter defibrillator. We received only 1 direct referral through our app despite supportive feedback. Specific reasons from our physician survey included the lack of integration into an EMR, as well as perceived efficiency of the current paper- or fax-based system. Future studies should continue to systematically evaluate smartphone apps in cardiology to assess their usability and feasibility and to assess the strategies for their integration into daily workflow.

## Abbreviations

EMR: electronic medical record
mHealth: mobile health
